# Supplementary material for: Clinical Practice of Targeted Capture Sequencing to Identify Actionable Alterations in Cholangiocarcinoma
Source: Cancers (Basel). 2022 Oct 16;14(20):5062. doi: 10.3390/cancers14205062 (PMC9600135; doi:10.3390/cancers14205062)
Supplement: Supplementary file 1 [file cancers-14-05062-s001.zip › Supplementary Figures.pdf]

Supplementary Figures

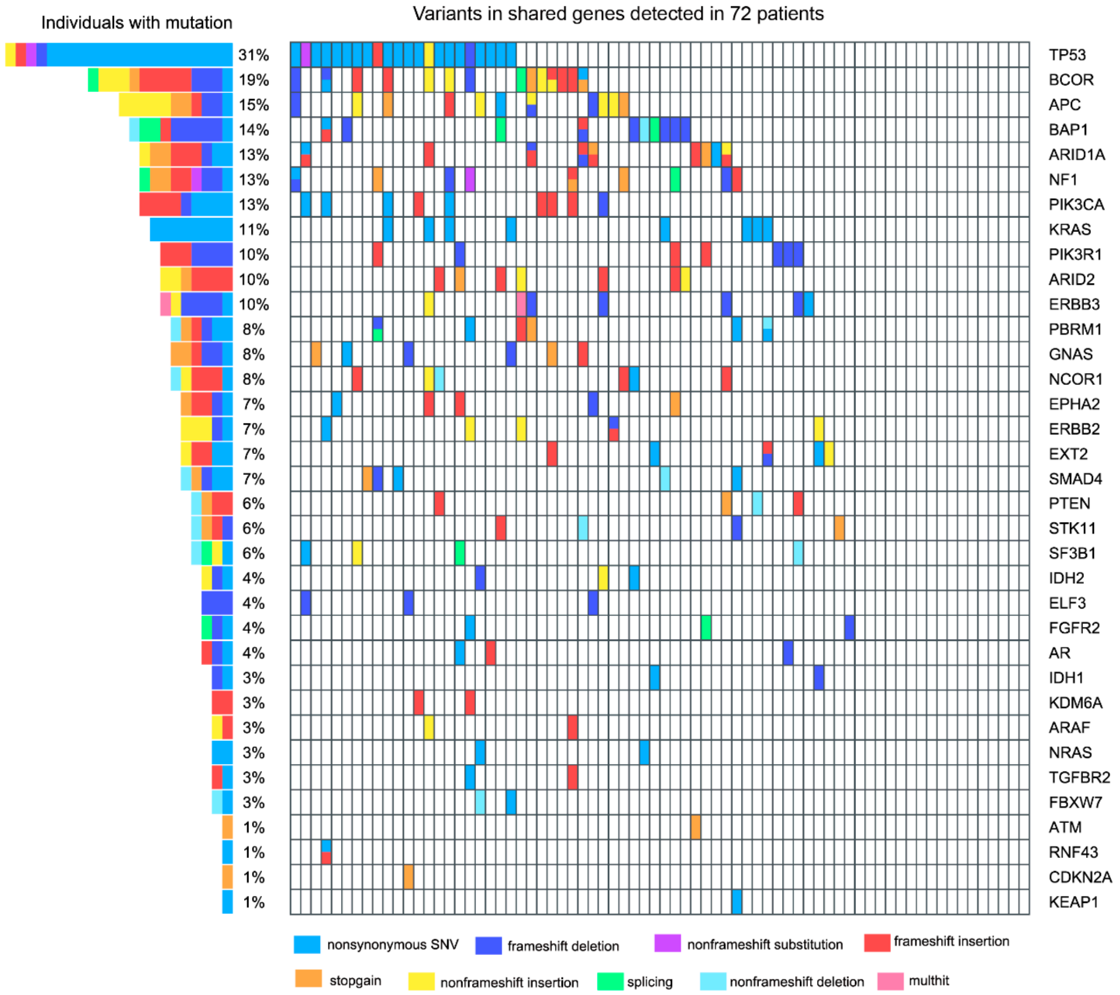

Figure S1. The landscape of all mutations in driver genes both detected by 508 and 688-gene panels in 72 patients. Left, the percentages of patients with mutations. Right, targeted genes are ranked based on the mutation frequency. Different colors correspond to different types of mutations. Variants annotated as Multhit are those genes that are mutated more than once in the same sample.

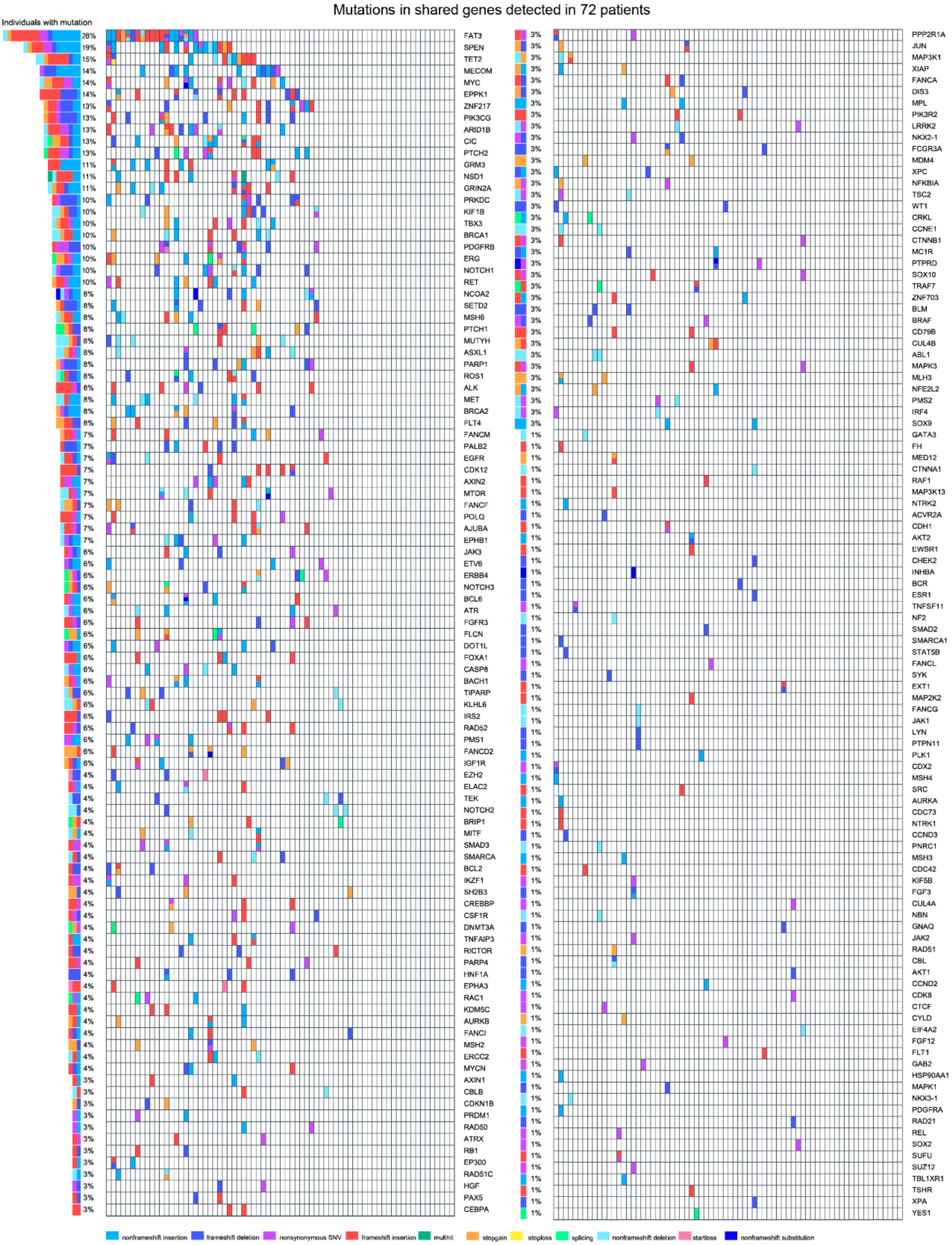

Figure S2. The landscape of all mutations in non-driver genes both detected by 508 and 688-gene panels in 72 patients.

Mutations in non-driver genes only detected by 688-gene panel in 63 patients

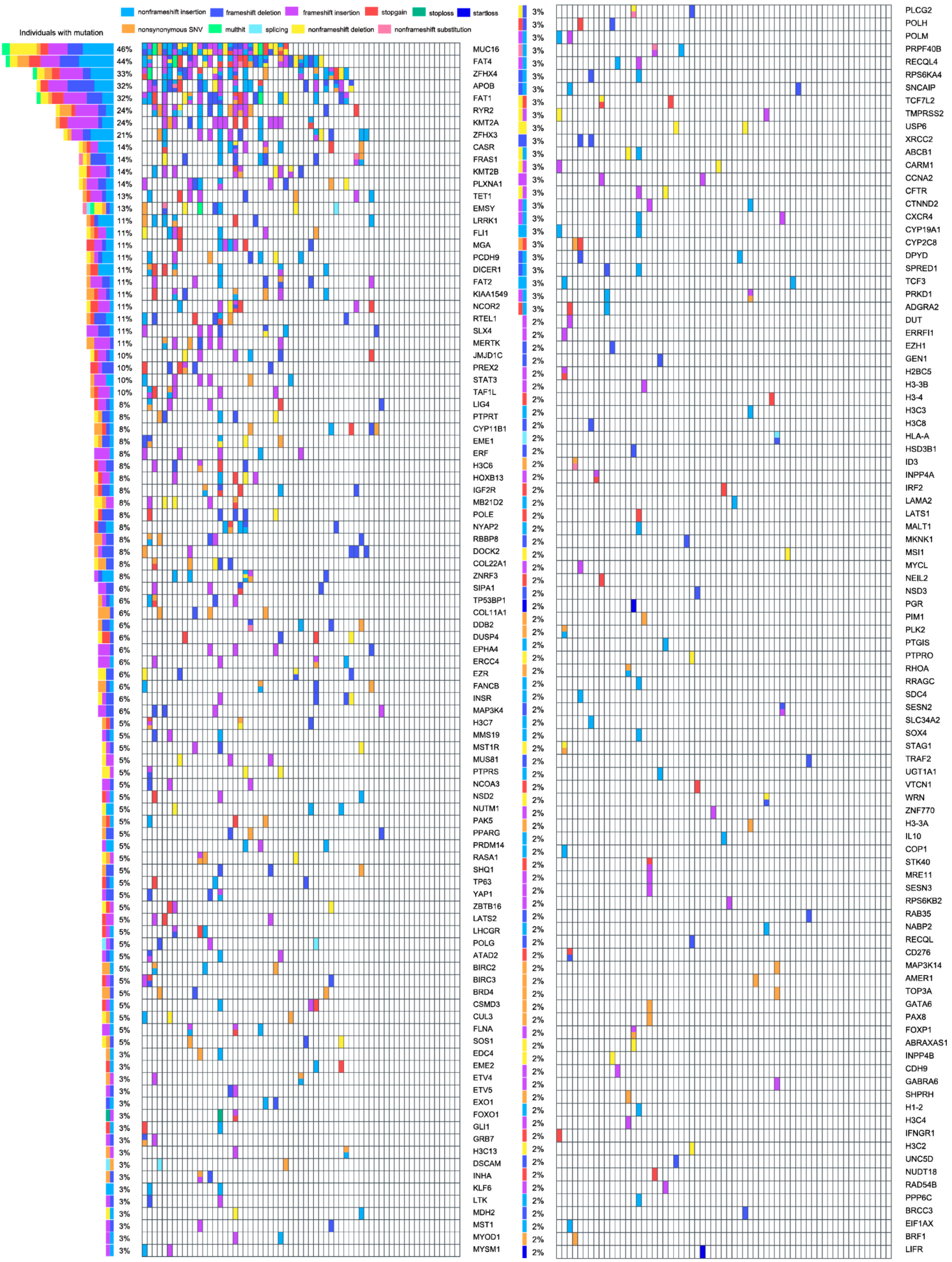

Figure S3. Mutation landscape of non-driver gene only detected by the 688-gene panel in 63 patients.

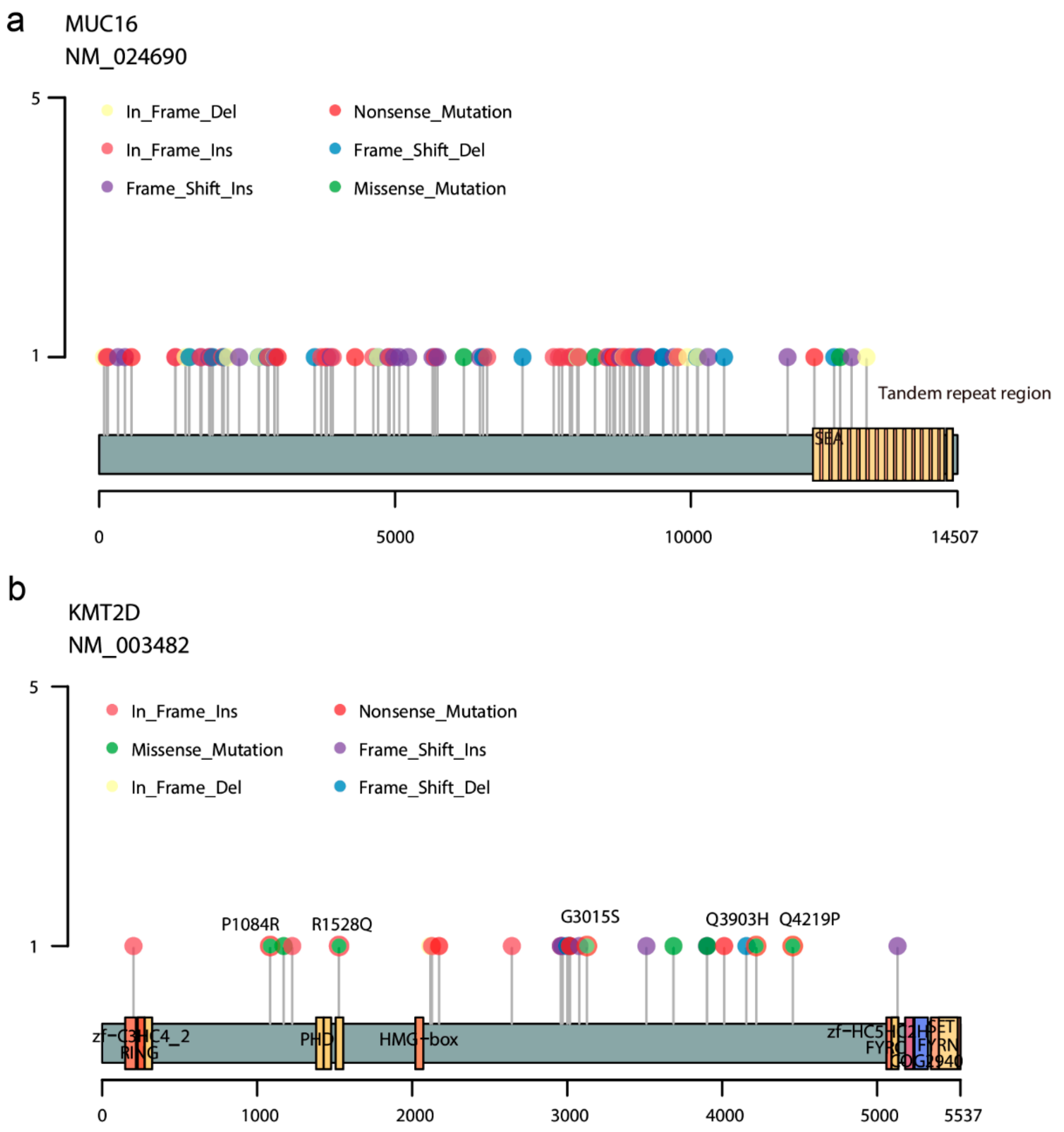

Figure S4. Location of the *MUC16* and *KMT2D* mutations schematic.

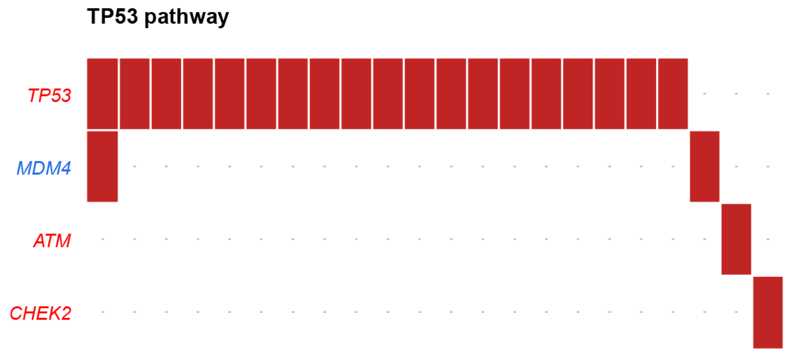

Figure S5. Mutated genes in the TP53 pathway. Tumor suppressor genes are in red, and oncogenes are in blue font.
